# The Gulf Coast: A New American Underbelly of Tropical Diseases and Poverty

**DOI:** 10.1371/journal.pntd.0002760

**Published:** 2014-05-15

**Authors:** Peter J. Hotez, Kristy O. Murray, Pierre Buekens

**Affiliations:** 1 Section of Pediatric Tropical Medicine, Department of Pediatrics, National School of Tropical Medicine, Baylor College of Medicine, Houston, Texas, United States of America; 2 Sabin Vaccine Institute and Texas Children's Hospital Center for Vaccine Development, Houston, Texas, United States of America; 3 James A. Baker III Institute for Public Policy, Rice University, Houston, Texas, United States of America; 4 Tulane University School of Public Health and Tropical Medicine, New Orleans, Louisiana, United States of America; University of Washington, United States of America

*The recent finding that dengue fever has emerged in Houston, Texas—the first major United States city in modern times with autochthonous dengue—adds to previous evidence indicating that the Gulf Coast of the Southern US is under increasing threat from diseases thought previously to affect only developing countries.*

**Figure 1:**
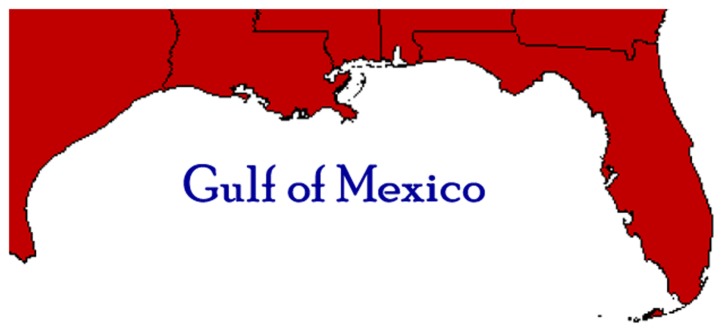
Gulf Coast of the US (original prepared by Nathaniel Wolf).

Extreme poverty and a warm, tropical climate are the two most potent forces promoting the endemicity of neglected tropical diseases in Africa, Asia, and Latin America. Now, these same forces are also widely prevalent in the five states of the US Gulf Coast—Texas, Louisiana, Mississippi, Alabama, and Florida (Figure 1). Poverty is rampant: ten million Gulf Coast residents currently live below the US poverty line, with Mississippi topping the list of all states in terms of percentage of people who live in poverty (22%) [1]. Texas alone has almost five million poor people [1]. Of particular concern is the level of extreme poverty—defined as less than one-half of the federal poverty level—in the region, especially among minorities. One in ten black children living in Louisiana and Mississippi live in such near-developing-nation-level conditions [2]. Superimposed on this pervasive extreme poverty are frequent and periodic exposures to climate and environmental hazards, including hurricanes, floods, droughts, and oil spills [3], [4], which in some cases can further exacerbate financial hardships in the region. Thus, today the Gulf Coast is currently considered America's most vulnerable and impoverished region [4], [5].

One of us (PJH) previously noted in 2011 how neglected tropical diseases could emerge in this mixing bowl of poverty and hardship in the Gulf (Table 1) [6]. At that time, the key factors linking poverty with disease on the Gulf Coast included housing with inadequate or absent plumbing, air conditioning, and/or window screens, and it was predicted that the region faces imminent threats from dengue fever and other vector-borne tropical infections [6]. Now, a new retrospective study of almost 4,000 sera samples has revealed that Houston, Texas, suffered from a seasonal outbreak of dengue fever caused by dengue virus type 2 (DENV-2) from May until September of 2003, with transmission (by *Aedes* mosquitoes) also occurring in the two subsequent years [7]. No information beyond this period is available, so it remains a possibility that dengue emerged prior to 2003 and might still be causing seasonal epidemics. Moreover, it was also reported that in 2004–2005 an outbreak of DENV-2 dengue fever occurred in Cameron County, more than 300 miles to the south on the Texas Gulf Coast [8], [9]. Additional news reports indicate that dengue returned to Cameron and Hidalgo Counties late in 2013. In both the Houston and South Texas outbreaks, the poorest communities were most affected [7]–[9].

**Table 1:** Actual or potential neglected tropical disease threats to the US Gulf Coast.

Disease	Previous 20th-century outbreaks or endemicity	Current 21st-century outbreaks or endemicity	Gulf Coast states known to be affected
**Viral Infections**			
Dengue fever	+	+	Texas and Florida
West Nile virus infection	-	+	All
St. Louis encephalitis	+	+	All
Chikungunya	-	-	None as of yet
Venezuelan equine encephalitis	+	-	Texas
**Bacterial Infections**			
Murine typhus	+	+	Texas
*Vibrio vulnificus*	+	+	All
**Parasitic Infections**			
Trichomoniasis	+	+	All
Toxoplasmosis	+	+	All
Chagas disease	+	+	Texas and Louisiana
Cutaneous leishmaniasis	+	+	Texas
Toxocariasis	+	+	All
Cysticercosis	+	+	Texas

In light of the locally acquired cases of dengue fever caused by DENV-1 in Florida in 2009–2010 [10], an added concern is whether the phenomenon of viral immune enhancement that could result from the presence of two different dengue serotypes (previous exposure to one serotype followed by infections with a different serotype) on the Gulf could place populations living there at future risk for dengue's most serious complications: severe dengue and dengue shock syndrome.

Beyond dengue, Texas previously suffered from regular St. Louis encephalitis summer outbreaks [11] and currently has had the largest number of cases of West Nile virus (WNV) infection (transmitted by *Culex* mosquitoes) of any state, with periodic spikes in the number of cases occurring at three-year intervals [12]. Possibly unique to WNV strains in Texas [13] is the observation that chronic persistent infection and prolonged immunoglobulin M (IgM) seropositivity is a common occurrence and is associated with several major clinical sequelae [14], including depression [15] and chronic kidney disease associated with viruria [16].

The US Gulf Coast is also considered vulnerable to the introduction of Chikungunya fever, an alphavirus infection transmitted by *Aedes* mosquitoes that clinically resembles dengue, with the possibility of year-round transmission in the warm Gulf climate [17]. Still another mosquito-transmitted viral infection—Venezuelan equine encephalitis (VEE)—spread rapidly from Guatemala and into Gulf coastal regions of Mexico and South Texas during the late 1960s and early 1970s, resulting in the deaths of 1,500 horses and several hundred human illnesses on the US side [18]. The VEE virus continues to actively circulate in areas of Mexico bordering the US [18].

Important neglected bacterial infections also stand out. Both murine and epidemic typhus have emerged among the homeless in Houston [19]. *Vibrio vulnificus* is a gram-negative bacterium of estuarine and coastal habitats of the northern Gulf of Mexico, where it has become an important opportunistic pathogen that can cause serious wound infections and primary septicemia among individuals who come into contact with seawater or contaminated seafood [20].

Among the parasitic infections now considered widespread in the Gulf Coast, trichomoniasis was shown to be the leading sexually transmitted infection and an important cofactor in the HIV/AIDS epidemic in New Orleans, Louisiana [6], [21]. Human autochthonous Chagas disease transmission has been confirmed in Texas and Louisiana [6], [22], [23]. Canine Chagas has also been found in these states. A recent economic analysis reveals that Chagas disease incurs almost $900 million in costs in the US [24], although the percentage of these costs for the Gulf region has not been specified. Similarly, toxocariasis, a soil-transmitted helminthic zoonosis, disproportionately occurs in the South, affecting as many as one in five non-Hispanic blacks and linked to low education levels and cognitive delays [25], but its prevalence in the Gulf is not known.

To date, the major social determinants of the neglected tropical diseases are poverty and also race or ethnicity. The actual biomedical underpinnings for these connections are poorly understood, although, with respect to poverty, in some cases poor housing may increase exposure to medically relevant vectors while lack of sanitation and access to clean water in impoverished areas, as well as lack of access to health care, would further promote disease. These diseases also disproportionately occur among non-Hispanic blacks and Hispanics, but this relationship may also be based mostly on links to poverty.

Still another observation is the association between some of these neglected tropical diseases and maternal and child health. There are an estimated 40,000 pregnant North American women who are *Trypanosoma cruzi* seropositive and at risk of transmitting the parasite to their babies [26]. Thus, there is an urgent need to measure the frequency of congenital Chagas disease and to evaluate the need for screening and treatment. Dengue in pregnancy is also increasingly recognized for its associations with increased risks of postpartum hemorrhage and preterm birth [27].

Some of the urgent needs in addressing the neglected tropical diseases in the Gulf have been summarized previously and include specific recommendations for greatly expanded disease surveillance and studies to determine exactly how these diseases are transmitted [6], [28], [29]. Currently, such studies are not being actively pursued across the Gulf region for any major neglected tropical disease. Mosquito control programs are often well organized, but there is a need to seriously investigate different control strategies for vector-borne diseases in order to reduce vector populations and host exposure [17]. For many neglected tropical diseases, diagnostic tests are cumbersome or not widely available. There is a severe lack of physician awareness about how to manage and treat neglected tropical diseases and an equally urgent need to develop new or better drugs and vaccines.

The stakes are high. The Gulf Coast remains vitally important to the American economy because of its key role in petrochemicals [3] and shipping [4]. Today, Houston and New Orleans represent two of the largest American ports [4], with expectations that these ports will continue to expand significantly with the imminent widening of the Panama Canal. Enhanced measures to detect, treat, and prevent neglected tropical diseases are important steps to promote the health of populations living on the Gulf and ensure the region's economic vitality.
